# Starling Hall

**Published:** 1855-05

**Authors:** 


### STARLING HALL—COLUMBUS, OHIO.

We have received numerous letters of enquiry, from time to time, in relation to institutions where the insane can be treated. As there is yet no Asylum erected in this State, and as some two years must elapse before the provision made by the last Legislature can be enjoyed, time will thereby be allowed for recent and curable cases to become confirmed, and perhaps incurable. Immediate provision should be made for such; but the enquiry will be, how this is to be accomplished. The States around us have their Asylums, and they have their unfortunate insane too, and in some of these States in greater numbers than they have adequate provision for. We must, therefore, turn to some private means or provision, and we know of none at present which promises so much as "Starling Hall," Columbus, Ohio, where Dr. Patterson has made comfortable arrangements for the care and treatment of a few insane females of other States than Ohio. Dr. Patterson was formerly Physician to the "Ohio Lunatic Asylum," and late Physician to the "Indiana State Hospital for the Insane." He has spent ten years in the treatment of insanity and nervous diseases; and, being an accomplished medical man, and a gentleman of philanthropy, we can most confidently recommend "Starling Hall" to those who desire a place for their unfortunate relatives or friends until arrangements can be made for them at home in our own State. Further information will be promptly given by application to Dr. Patterson, at "Starling Hall," or the Editors of this Journal.

